# Patient perspectives on life impact and unmet needs in giant cell arteritis and polymyalgia rheumatica: insights from social media

**DOI:** 10.1093/rap/rkaf140

**Published:** 2025-12-02

**Authors:** Sarah L Mackie, Pallavi Arun, Vandana Padmanabhan, Alvaro Arjona, Joyce A Kullman

**Affiliations:** Leeds Institute for Rheumatic and Musculoskeletal Medicine, University of Leeds, Leeds, UK; NIHR Leeds Biomedical Research Centre, Leeds Teaching Hospitals NHS Trust, Leeds, UK; Novartis Healthcare Private Limited, Hyderabad, India; Novartis Healthcare Private Limited, Hyderabad, India; Novartis Farmacéutica, S.A., Madrid, Spain; Vasculitis Foundation, Kansas City, MO, USA

**Keywords:** patient perspectives, glucocorticoids, polymyalgia rheumatica, giant cell arteritis, quality of life

## Abstract

**Objective:**

To ascertain the perspectives of caregivers and patients with giant cell arteritis (GCA) and/or polymyalgia rheumatica (PMR) and the impact of disease on quality of life (QOL) and identify their unmet needs through social media listening.

**Methods:**

This retrospective study analysed social media posts from the USA and Germany between 1 August 2022 and 1 August 2023. A keyword search query retrieved posts addressing key research questions from various social media platforms using the Sprinklr tool. Natural language processing was used to assess the relevance of the posts, followed by a manual analysis to identify themes, map key topics and derive insights.

**Results:**

Of 1001 unique posts, 517 were from patients or caregivers; these yielded 919 references to specific topics. Posts about PMR (65%) were more frequent than those about GCA (18%) or both (17%). The most common topics discussed by patients and caregivers were related to treatment (33%), symptoms (13%), healthcare professional (HCP) visits (10%), diagnosis (7%) and impact on QOL (7%). Among the mentions expressing treatment-related sentiment (*n* = 297), negative sentiment was predominant (61%) across PMR- and GCA-related posts. Glucocorticoids were perceived more negatively than biologics. Patients reported significant impact of the disease on their QOL. Unmet needs were mostly related to experiences with HCPs and treatments.

**Conclusion:**

This novel social media listening study provides insights into the lived experiences of GCA and PMR. Patient conversations revealed multiple impacts on QOL and reflected a need for more effective, better-tolerated treatments and for greater disease awareness among healthcare professionals.

Key messages

- This is the first social media listening-based study to report insights from PMR and GCA patients.
- PMR and GCA patients reported a substantial quality-of-life impact and the need for increased disease awareness.
- PMR and GCA patients expressed negative sentiment towards current treatments, highlighting the need for effective glucocorticoid-sparing therapies.

## Introduction

Giant cell arteritis (GCA) and polymyalgia rheumatica (PMR) are systemic inflammatory diseases that may occur asynchronously or concurrently, affecting adults >50 years of age [1, 2]. Clinically, 40–60% of patients with GCA have PMR symptoms at diagnosis. Nearly 16–21% of patients with PMR are also diagnosed with GCA before, during or after a PMR diagnosis [3, 4]. Women are affected two to three times more frequently than men [3].

GCA is the most common type of primary systemic vasculitis, affecting medium- and large-sized arteries. GCA symptoms include headache, scalp tenderness, jaw claudication and visual impairment [2, 5]. GCA should be diagnosed and treated promptly to avert the risk of visual loss; diagnostic delay may lead to ischaemic complications such as visual loss or stroke [6]. PMR is the second most common inflammatory rheumatic disease in patients >50 years of age and is diagnosed clinically [7]. The characteristic symptoms of PMR are pain and stiffness in the neck, shoulders, upper arms, pelvic girdle, hips and thighs [8]. Systemic manifestations such as low-grade fever, sweats, loss of appetite or weight loss occur in 40–50% of patients with PMR [1, 9, 10] and are also common in GCA. Fatigue and depression are common in both conditions [10, 11].

Most patients with PMR and GCA have two or more comorbidities, which may interact with the disease or treatment [12, 13]. Glucocorticoids (GCs), which remain the mainstay treatment for PMR and GCA, may cause or worsen many of these comorbidities. After remission is achieved, it is recommended to taper the GC dose. However, relapse commonly occurs during GC tapering, necessitating dose re-escalation [14–16]. Given the chronic nature of these diseases and their tendency to relapse, patients may require long-term treatment leading to high cumulative doses of GCs, resulting in GC-related toxicity [17–19].

Traditional research methods used to study patients’ views and experiences, such as interviews, surveys and patient focus groups, have limitations, including selection, recall and reporting bias [20]. Patients increasingly use social media to share their disease experiences [21, 22]. In contrast to traditional research methods, social media listening provides access to first-person, authentic, spontaneous, unbiased and unfiltered perspectives of patients on their disease experiences, treatment and unmet needs [23]. Social media can help patients by supplementing information from healthcare professionals (HCPs) [24]. A systematic review has shown that patients do not use social media to circumvent HCPs but rather use it to fill knowledge gaps and to complement information provided by HCPs [25]. Furthermore, patients may join online health communities to seek emotional support and to understand lived experiences that differ from those typically provided by their HCPs [25]. Recent studies suggest that patients using social media for disease-related information felt empowered and communicated better with HCPs [26]. Previous studies have shown that social media is valuable for understanding patient experiences with rheumatic diseases [27–30].

Currently there are no published social media listening studies evaluating the impact of PMR and GCA on the quality of life (QOL) of patients and their unmet needs. Analysing social media conversations of patients and caregivers can provide useful insights into how patients discuss their conditions online; understanding and appreciating these narratives may help physicians understand how to better meet patient needs [21]. In this study we aimed to use social media listening to ascertain patients’ and caregivers’ perspectives on PMR and GCA, including their unmet needs and the impact of disease on their QOL.

## Methods

**Figure 1:**
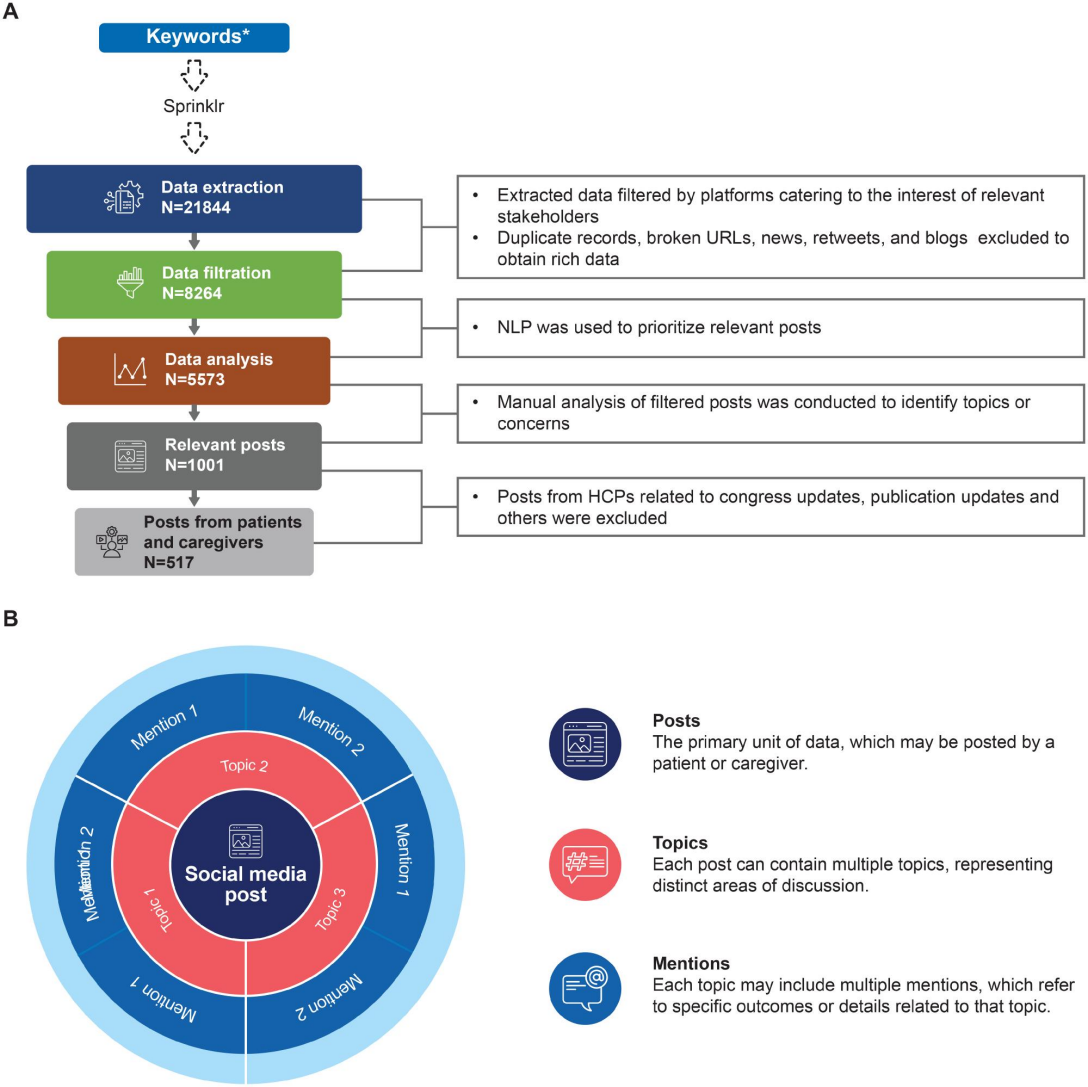
Data collection and methodology. **(A)** The methodology of the social media listening study and **(B)** hierarchical relationships between posts, topics and mentions. Social media posts related to GCA and PMR discussions from the USA and Germany were extracted using the Sprinklr tool. Social media content was filtered by platforms and analysed using natural language processing followed by manual review to extract key insights from patients’ and caregivers’ posts. Each unique ‘post’ may include multiple ‘topics or concerns’ and each of these ‘topics’, in turn, may entail multiple ‘mentions’ referring to specific outcomes related to the topic. ^a^Keyword search criteria are included in Supplementary Data S1, available at *Rheumatology Advances in Practice* online. NLP: natural language processing; URL: uniform resource locator

This was a retrospective study based on public social media posts. A literature review was used to formulate search query for GCA and PMR (included in Supplementary Data S1, available at *Rheumatology Advances in Practice* online) to retrieve the online content that addressed the key research questions. The Insights tool (Sprinklr, New York, NY, USA) was used to download publicly available social media posts, using a broad search query for GCA and PMR, from platforms such as X, Instagram, Facebook, forums and blogs. Social media content originating from the USA and Germany in both English and German were extracted for the period 1 August 2022–1 August 2023 (Fig. 1A). Additionally, secondary research was performed by manually reviewing forums for discussions among patients and caregivers.

The extracted data were filtered by platforms catering to patients, caregivers and HCPs. Duplicate records, broken links, news, retweets and blogs were excluded. Natural language processing was used to assess the relevance of the posts, followed by manual review to identify key topics and derive insights. Demographic details and diagnosis were recorded when available or marked as unknown. Assumptions about patients’ experiences were avoided unless explicitly stated in the posts.

A post refers to an anonymized online discussion by a patient or caregiver and is the primary unit of data. The terms ‘post’ and ‘conversation’ are used interchangeably. The number of posts does not directly correspond to the number of patients or caregivers, as the same patient may have produced multiple posts. Each unique ‘post’ may include multiple ‘topics’ and each ‘topic’, in turn, may entail multiple ‘mentions’ referring to specific outcomes related to the topic (Fig. 1B). Considering that each post is unique and has been objectively analysed to address research questions, there is no duplication of posts, eliminating the possibility of double counting.

## Data analysis

All data were analysed using descriptive statistics and are presented as the number of posts, number of topics and number of mentions or their percentages. As one post can encompass multiple mentions, the number of ‘mentions’ has been used as the basis for calculating percentages of the outcome under each topic, unless stated otherwise.

## Ethical considerations

This study does not contain any identifiable participants. All web-based content was anonymized and was in accordance with the Health Insurance Portability and Accountability Act search strategy and data collection. Approval was obtained from the Novartis safety registry 1P1R, the governing body that holds oversight on the use of social media by Novartis (DE014165).

## Results

### Patient demographics and topic landscape

Among 1001 unique social media posts on PMR and GCA, 517 posts were from patients and caregivers, of which 90% originated from the USA. Among the 105 posts mentioning patient sex, 65% were female. Of the 43 posts reporting age, most patients were ≥60 years.

Posts on PMR (65%) were more frequent than those on GCA (18%), while 17% of the posts involved both conditions. Among them, 2% each for PMR or GCA were related to suspected diagnosis. The most commonly used social media platforms by patients and their caregivers were X (formerly Twitter) and forums including Reddit and Quora. The various forums that were mostly used by patients and caregivers included forum.rheuma-online.de and psychic.de in Germany and Reddit, Quora, connect.mayoclinic.org, community.myfitnesspal.com and scribble.freeforums.net in the USA.

A total of 517 posts from patients and caregivers had 919 references to specific topics. The most common topics discussed were related to treatment (33%), symptoms (13%), HCP visits (10%), diagnosis (7%) and impact on QOL (7%). The other topics were regarding causes and triggers (6%), comorbidities (6%) and disease management (4%).

These topics were grouped into five categories for the purpose of this article, namely treatment-related patient sentiments, disease-related patient experiences, HCP-related patient experiences, impact on QOL and unmet needs.

### Treatment-related patient sentiments

Among the 304 posts discussing various treatment options for PMR and GCA, there were 297 mentions expressing patients’ sentiment toward treatments, of which, 61% (*n* = 181) expressed a negative sentiment on various treatments. The proportion of negative sentiment mentions coming from PMR and GCA posts was 52% and 19%, respectively, likely reflecting the disease distribution in the analysis set.

Of the 121 mentions in PMR-related posts that expressed a sentiment on GCs, 57% reflected a negative sentiment. Similarly, of the 32 mentions in GCA-related posts, 72% expressed a negative sentiment towards GCs. The treatment-related mentions found in posts coming from patients with both PMR and GCA predominantly expressed a negative sentiment towards GCs (75% of 44 mentions) and biologics (71% of 17 mentions).

**Figure 2:**
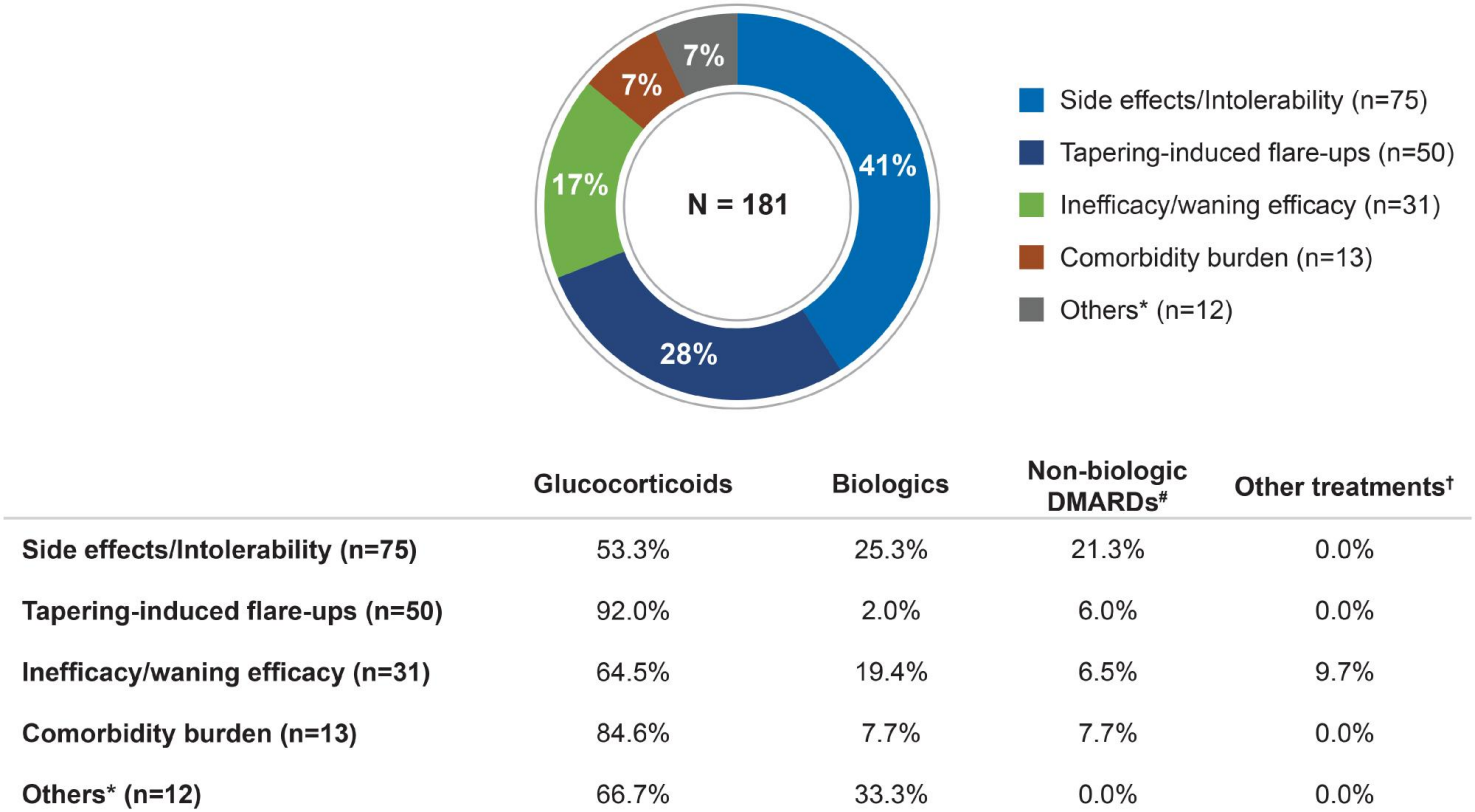
Negative sentiment drivers across treatment modalities in PMR and GCA. The pie chart presents the frequency of negative sentiment drivers for treatments based on the analysis of social media posts by patients with GCA and PMR and caregivers. The table shows the frequency of treatment modalities (GCs, biologics, non-biologic DMARDs and others) in each negative sentiment category. *N*: number of mentions related to negative sentiments; *n*: number of mentions related to negative sentiment drivers. ^a^Others include ‘treatment for a pre-existing condition makes it difficult to track progression’ and ‘non-compliance’. ^b^Non-biologic DMARDs include methotrexate and leflunomide. ^c^Other treatments include, gabapentin NSAIDs, tofacitinib and anti-inflammatory drugs

Negative sentiments resulted from side effects/intolerability, inefficacy/waning efficacy, flares during GC tapering and comorbidity burden (treatment causing/exacerbating comorbidities). Among 75 mentions referring to side effects/intolerability, GCs were implicated in 53% and biologics in 25%. Tapering-induced flare-ups (*n* = 50) were predominantly associated with GCs (92%). Among 31 mentions of inefficacy or waning efficacy, 65% were related to GCs and 19% to biologics (Fig. 2).

Overall, only 39% of treatment-related mentions (*n* = 297) reflected a positive sentiment. PMR-related posts contributed 65% of these, while GCA-related posts accounted for 20%. Sentiment on biologics was positive in both PMR (64% of 28 mentions) and GCA posts (59% of 22 mentions).

Positive sentiments resulted from efficacy, safety and tolerability, remission, no tapering-related issues and positive impact on QOL. Specifically, of the 54 mentions on efficacy, 70% were on GCs and 26% on biologics. Among the mentions on successful tapering (*n* = 17), 41% were attributed to GCs and 47% to biologics. Of those referring to safety and tolerability (*n* = 14), 50% were on GCs and 29% on biologics. Furthermore, among the mentions regarding the attainment of remission (*n* = 13) and the positive impact on QOL (*n* = 9), 92% and 78% of the mentions, respectively, were associated with GC use.

### Disease-related patient experiences

Posts from patients and caregivers discussed various disease-related topics, including causes and triggers, comorbidities, symptoms and diagnosis. Patients reported that they experienced heterogeneous symptoms and comorbidities, often requiring consultations with multiple HCPs before a confirmatory diagnosis was made.

**Figure 3:**
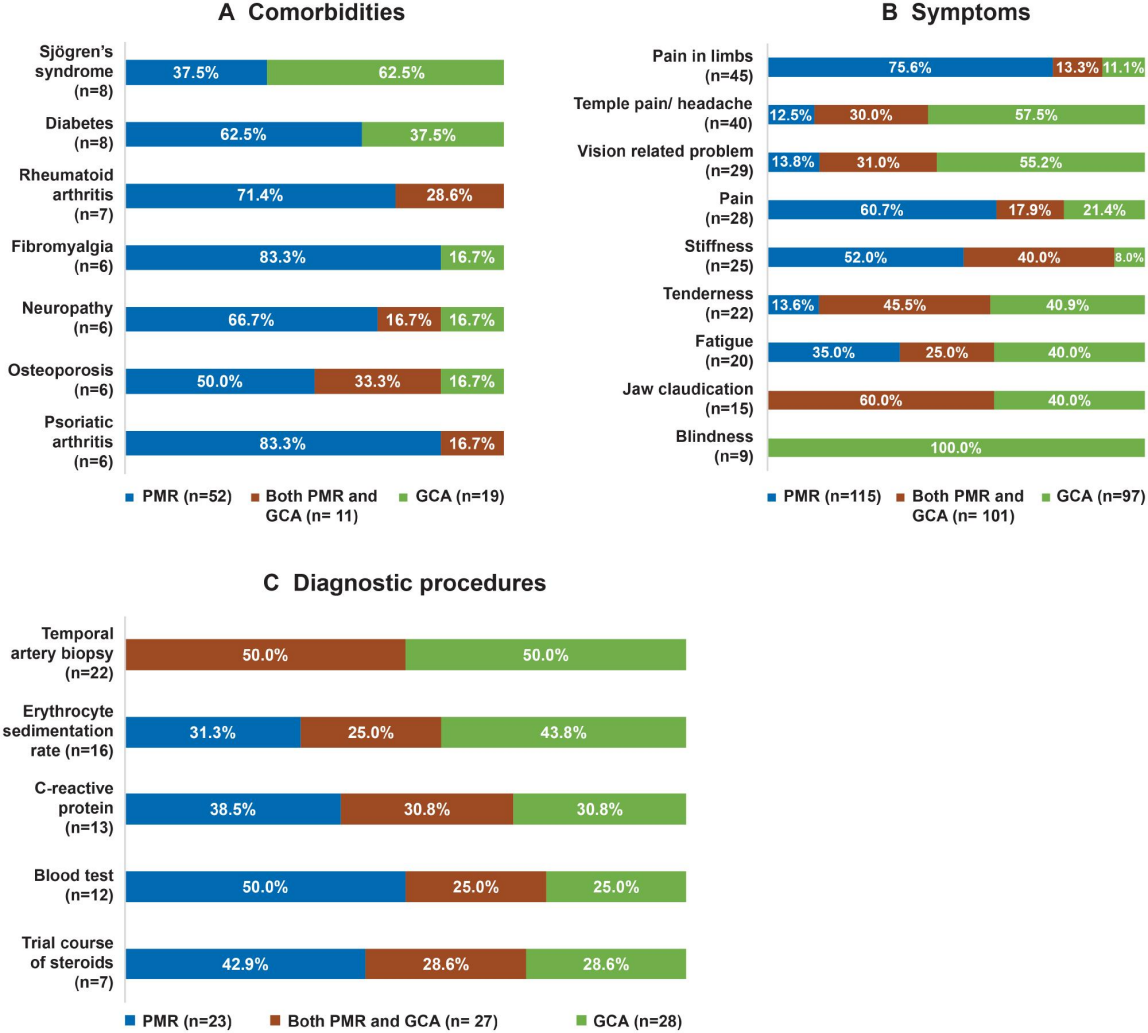
Common comorbidities, symptoms and diagnostic procedures mentioned in online posts by PMR and GCA patients and caregivers. Only common comorbidities and symptoms reported in at least five unique conversations related to GCA and PMR are included. *n*: number of mentions. Percentages on the table refer to the proportion of mentions coming from PMR- or GCA-related posts. In the overall analysis set, 65% of posts were PMR related, 18% were GCA related and 17% were related to both diseases. Due to rounding, some percentages do not sum to 100%

In total, 59 posts included 64 mentions regarding the causes and triggers of PMR and GCA. Where causes or triggers were attributed, the most frequently mentioned was COVID infection or COVID vaccine (69%). Posts from patients also suggested that work-related stress and/or cold weather (14%), family history and genetic predisposition (6%), vaccination against shingles (6%), flu vaccination (3%) or hormonal changes (2%) could have triggered their condition. Furthermore, 54 posts included 121 mentions of comorbidities, with diabetes (*n* = 8), Sjögren’s disease (*n* = 8) and RA (*n* = 7) as the most frequently mentioned conditions (Fig. 3A).

There were 329 mentions related to GCA and PMR symptoms discussed in 122 posts by patients and caregivers. The symptom profiles of PMR and GCA were largely typical for these conditions. Posts by PMR patients complained of severe morning stiffness, limb pain and fatigue as their most bothersome symptoms. Further, posts by GCA patients included severe headache/temple pain, visual symptoms and jaw pain (Fig. 3B). Among the 164 mentions related to emotional aspects, feeling anxious (46%), confused (30%), helpless (14%) and frustrated (10%) were the most cited emotions.

There were 78 mentions related to diagnosis among 60 posts; commonly mentioned diagnostic procedures included temporal artery biopsy (TAB), ESR, CRP and ‘blood tests’ (Fig. 3C). TAB was the most frequently mentioned diagnostic procedure for GCA. Only a few patient posts mentioned imaging, and ultrasound was not mentioned at all. The diagnostic delay ranged from <6 months in a few cases to >6 years in others. Some patient posts reported normal blood tests despite the presence of symptoms, leading to delayed diagnosis. Additionally, posts from patients mentioned that GCA/PMR was initially misdiagnosed due to symptoms that overlapped with other conditions such as temporomandibular joint dysfunction, sinus infection, fibromyalgia, tension headache and migraine.

### Experiences with HCPs

There were 18 mentions related to first contacts with HCPs and onward referrals. Most patient posts mentioned that they first visited ‘doctors’ (unspecified specialty, 44.4%), followed by ‘emergency room doctors’ (22.2%). Mentions of HCP referrals were most common to rheumatology, followed by ophthalmology. Reasons for referrals included the need to confirm the diagnosis and being unsatisfied with the primary care physician.

Of the 92 posts, 159 mentions described patient–HCP interactions, including both positive (40%) and negative (60%) experiences with HCPs. Negative experiences included a lack of empathy, long waiting periods, delayed diagnosis and misdiagnosis. Mentions of positive experiences included attributes such as empathy, active listening, proper attention, timely referrals, monitoring comorbidities, care and cooperation.

### Impact on QOL

**Figure 4:**
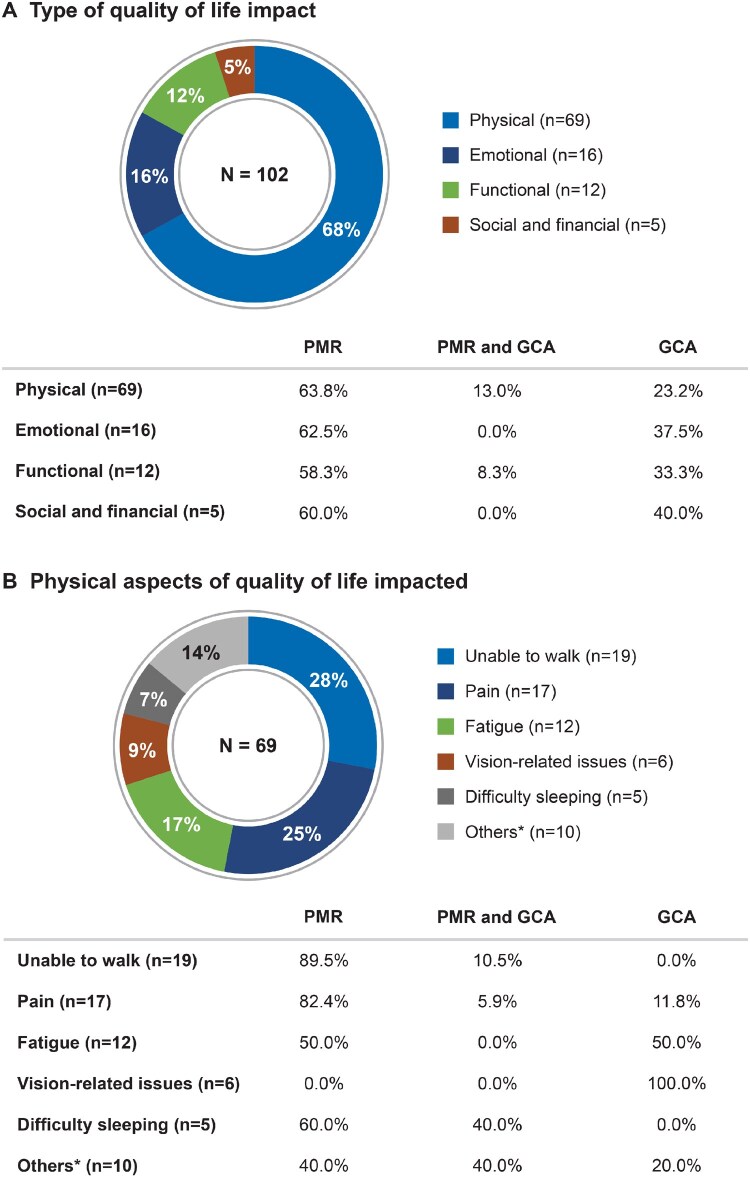
QOL impact as described in online posts by PMR and GCA patients and caregivers. **(A)** Pie chart presents the frequency of various aspects of QOL impacted by PMR and GCA, based on social media post analysis. *N*: number of mentions related to QOL. **(B)** Pie chart presents the frequency of various physical aspects of QOL impacted by PMR and GCA based on social media post analysis. ^a^Others include hospitalization, difficulty chewing, joint pain, loss of appetite, weight loss and comorbidity burden. *N*: number of mentions related to the impact on physical aspects of quality of life. Percentages on the table refer to the proportion of mentions coming from PMR- or GCA-related posts. In the overall analysis set, 65% of posts were PMR related, 18% were GCA related and 17% were related to both diseases. Due to rounding, some percentages do not sum to 100%

A total of 102 mentions across 60 posts described the impact of GCA and PMR on QOL. Among those reporting the impact of their disease on physical QOL (*n* = 69), nearly 64% were from PMR and 23% were from GCA posts (Fig. 4A), proportionate to the disease distribution in the dataset. However, the nature of the impact on physical QOL differed between the two conditions, with mentions of pain, difficulty walking and sleeping predominating in PMR-related posts and visual symptoms and fatigue in GCA-related posts (Fig. 4B).

Among the mentions related to the impact on QOL in relation to everyday functioning (*n* = 12), patients described how symptoms such as pain hindered their ability to perform daily activities and led to reduced productivity at work.

The mentions regarding emotional impact on QOL (*n* = 16) in patient posts were mostly attributed to physical symptoms such as chronic pain and functional impairment. Among the mentions related to the impact on QOL due to depression (*n* = 7), 71% were from PMR and 29% from GCA posts. Anxiety (*n* = 4) was reported to have impacted patients’ QOL in PMR- and GCA-related posts. Patient posts cited the unpredictable nature of the disease and GC tapering as the cause of anxiety. Some posts mentioned patients being in shock (*n* = 3) after their diagnosis of PMR or GCA.

Some patients’ conversations also mentioned that restricted movement and inability to work due to pain further impacted them financially and socially (*n* = 5), along with concerns about the lack of empathy from others.

### Unmet needs

**Figure 5:**
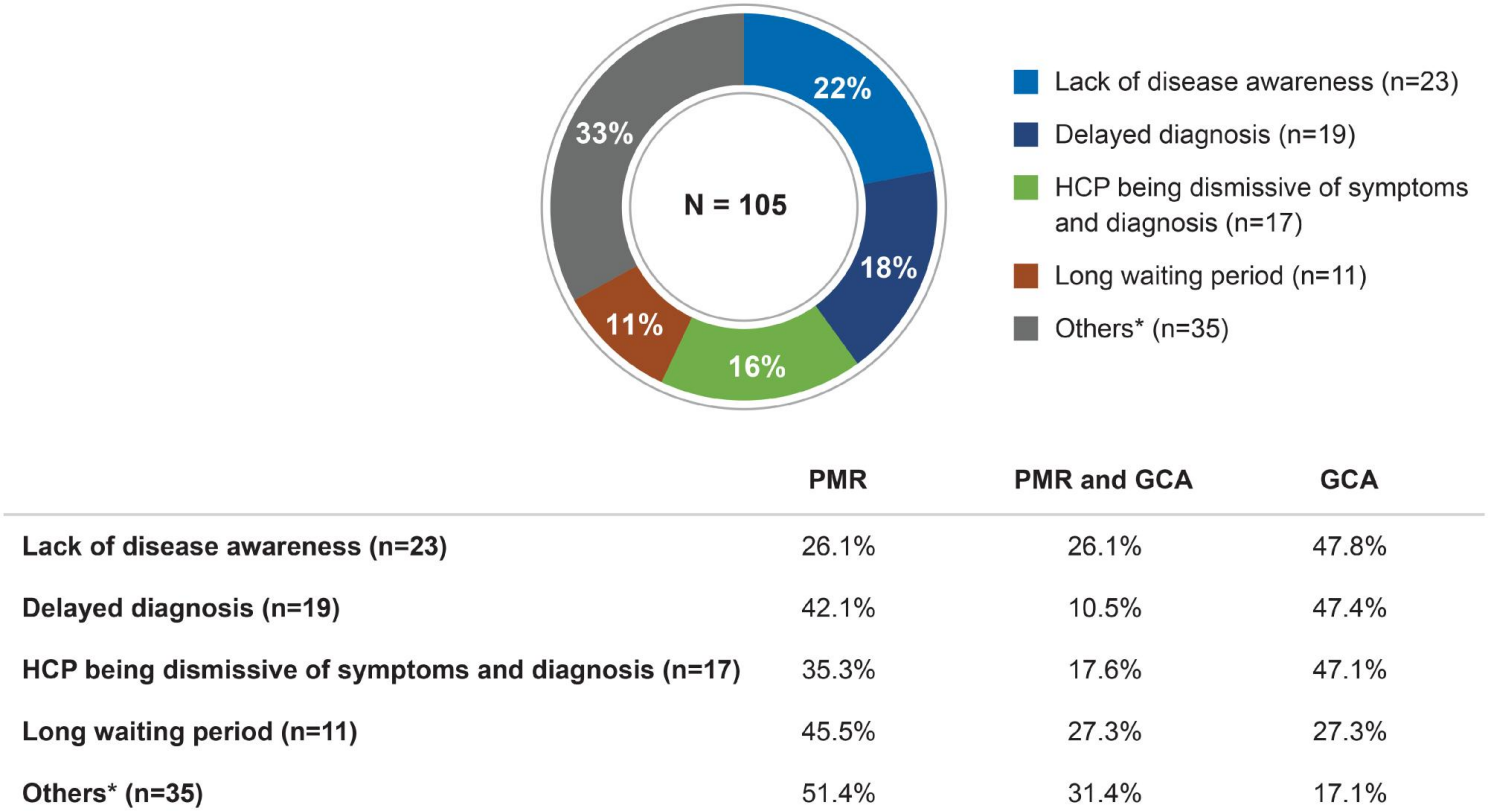
Care-related unmet needs as described in online posts by PMR and GCA patients and caregivers. Pie chart presents the care-related unmet needs of patients with PMR and GCA based on social media post analysis. *N*: number of mentions concerning care-related unmet needs. Percentages on the table refer to the proportion of mentions coming from PMR- or GCA-related posts. In the overall analysis set, 65% of posts were PMR related, 18% were GCA related and 17% were related to both diseases. Due to rounding, some percentages do not sum to 100%. ^a^Others include lack of consideration for a patient’s treatment choices, misdiagnosis, HCP did not provide adequate information, lack of empathy from HCPs and lack of confidence in HCP consultation

A total of 200 posts generated 250 mentions related to unmet needs, of which 105 mentions (42%) were related to medical care. Overall, these came disproportionately from GCA posts when compared with PMR posts when considering the overall posts on disease distribution. These conversations indicated that patients perceived that their HCPs lacked disease awareness and did not provide adequate information about their condition, its causes and treatment options. Patient posts also mentioned that HCPs were dismissive of patients’ symptoms and did not take their treatment preferences into consideration (Fig. 5). Among the 19 mentions related to diagnostic delays, 47% involved delayed referrals by ophthalmologists and ER doctors in GCA patients, resulting in visual symptoms. Patient posts mentioned long waiting times for HCP appointments, with GCA patients expressing concerns that their condition might deteriorate and impact their vision before they could see a specialist.

**Figure 6:**
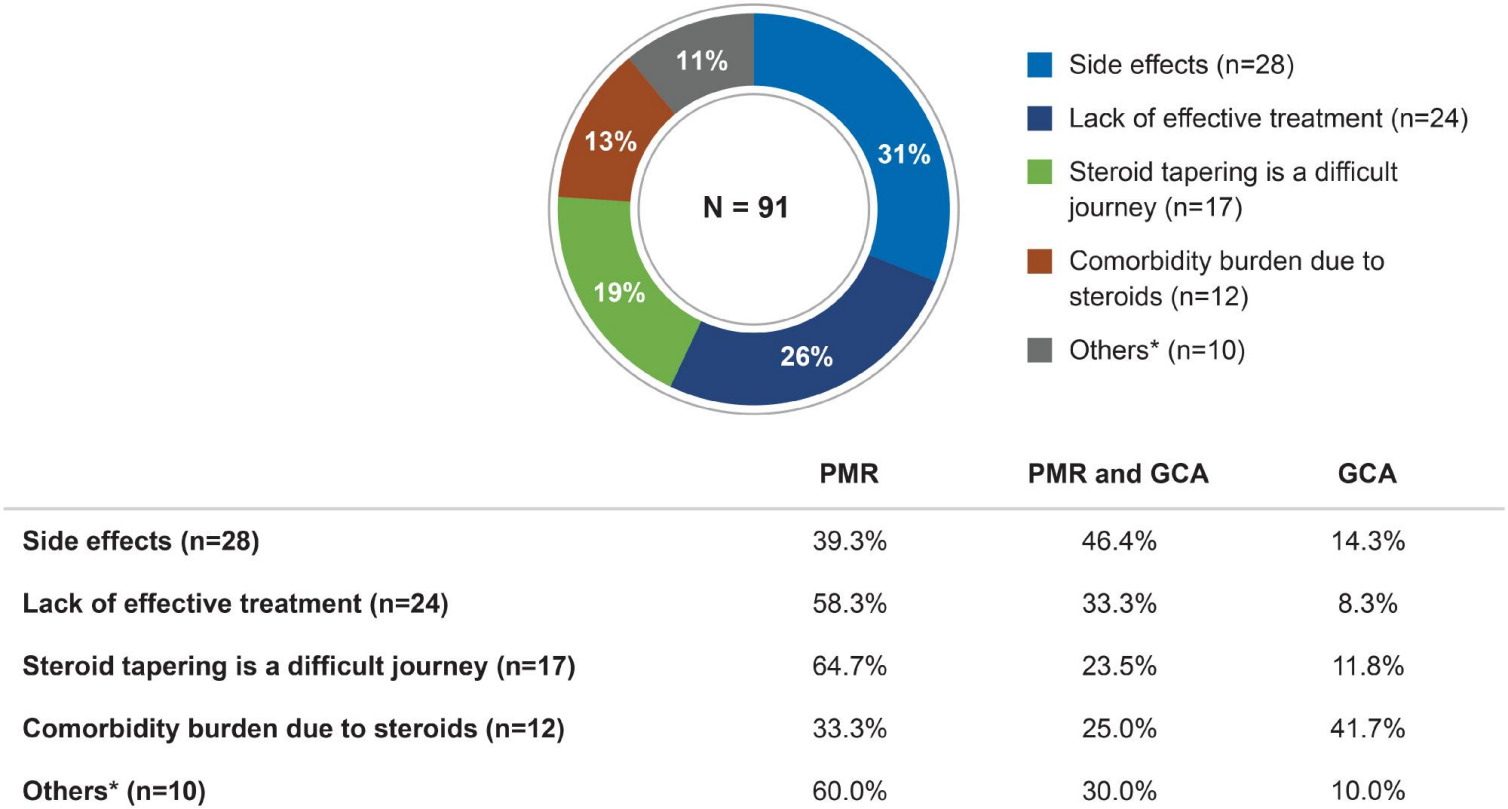
Treatment-related unmet needs as described in online posts by PMR and GCA patients and caregivers. Pie chart presents the treatment-related unmet needs of patients with PMR and GCA based on social media post analysis. *N*: number of mentions concerning the treatment-related unmet needs. Percentages on the table refer to the proportion of mentions coming from PMR- or GCA-related posts. In the overall analysis set, 65% of posts were PMR related, 18% were GCA related and 17% were related to both diseases. ^a^Others include self-GC tapering, cost/insurance, lack of information and treatment overshadowing symptoms

There were 91 mentions (36%) highlighting treatment-related unmet needs. Patient posts reported side effects such as insomnia, high blood sugar and rapid weight gain with long-term GC use. Generally, a desire toward rapid GC dose tapering due to side effects was expressed in patient posts. However, tapering was often mentioned as challenging due to the lack of other available treatments (Fig. 6). Patient posts also raised concerns about short-term symptom relief and frequent flares, which contributed to the overall disease burden. Nearly 8% of patient queries (*n* = 86) sought information about peer experiences before initiating the treatment.

Additionally, there were 29 mentions of disease-related unmet needs expressed in GCA and PMR patient posts. These included impacts on QOL and ability to perform daily activities, difficulties identifying disease progression and flare-ups due to overlapping symptoms of comorbidities and having insufficient information about causes, symptoms and other aspects related to their condition.

There were 25 mentions (10% of the unmet need mentions) that highlighted diagnosis-related unmet needs. Patient posts frequently indicated that diagnostic delays were often due to misleadingly normal test results, especially blood tests for PMR and TAB for GCA. In many cases, the normal results of these tests at the time the test was performed were attributed to receiving steroid treatment.

## Discussion

To the best of our knowledge this is the first social media–based study exploring the perspectives of patients with PMR and GCA and their caregivers. The free-text analysis in this social media listening study offers a unique approach for directly eliciting patient and caregiver experiences on a greater scale than traditional qualitative studies, without some of the limitations of survey-based approaches. Instead, our findings reflect what patients and caregivers say when they talk to each other rather than to HCPs or researchers.

Regulatory bodies encourage patient-focused initiatives to accommodate patient voices in the drug development process. As a source of real-world evidence, social media conversations have the potential to capture a wide range of authentic, spontaneous and unfiltered conversations about PMR and GCA among patients and the public. Social media listening is an observational method where the researcher analyses conversations that arise spontaneously. Unlike the traditional methods of surveys, interviews or patient focus groups, social media listening avoids biases that might arise by asking a question in a specific manner (leading-question bias). Online social platforms can thus provide important insights into the patient journey as experienced by the patients and caregivers [23]. Social media has a large population coverage, including remote and rural areas and various underserved groups that may face barriers to participation in research or care [23]. Patient-generated health data from social media can provide additional information for monitoring health outcomes and drug safety, as well as capturing symptoms or experiences that might otherwise go unrecorded [31].

Many platforms are designed to create ‘community’ by promoting posts to individuals with shared common interests. Due to network effects, highly connected, prolific and/or visible accounts can significantly influence the discourse around a disease. Therefore the quantitative data derived from these platforms should not be taken as representative of those from the entire patient population. Nevertheless, it is important for physicians to understand the social context that shapes patients’ knowledge, just as the medical literature informs physicians’ understanding. Physicians are often unaware of the online narratives influencing patients’ views of their disease and treatment.

Despite a diverse range of background comorbidities, the symptoms and impact described by patients with PMR/GCA were largely in line with clinical descriptions of these diseases. Visual symptoms were common; nine patients with cranial GCA presented with blindness. ‘Invisible’ symptoms such as pain, fatigue and brain fog impaired not only activities of daily living but also work. These findings are supported by those from previous qualitative research into the symptoms and life impact of PMR/GCA [11, 32]. Patients often enquired about the causes or triggers of their condition; attribution to COVID-19 or COVID vaccine was common, perhaps reflecting the time period during which these data were collected. Identifying causes or triggers could be one way by which patients deal with the uncertainty associated with the difficult journey of medication tapering and the fear of future relapses.

Patient experiences of delayed diagnosis or ambiguous/contradictory test results might have reduced trust in HCPs or contributed to perceptions that HCPs’ knowledge about PMR/GCA is insufficient. The dyadic idea of ‘shared decision-making’ as taking place between HCP and patient does not account for the digitally networked aspects of patients’ lives or their use of social media to seek experiences and stories of others. A survey conducted in a tertiary care hospital showed 70% of patients with rheumatic diseases acquired medical information through social media [33]. Similarly, in our study, some patients seemed to be seeking others’ opinions about treatment experience before initiating treatment themselves. This extended model of shared decision-making has been facilitated by social media and is an important trend that physicians should be aware of.

The majority of the posts on treatment-related sentiments were negative, except for biologics, which had more positive sentiments than negative. Indeed, the number of mentions of biologics in relation to both PMR and GCA was surprising given that most prescribing data still indicate that biologics are currently used in a minority of patients with GCA/PMR [34, 35]. This aligns with a previous social media–based study in RA, where sentiment analysis indicated that biologic DMARDs were perceived positively by patients with RA [36]. In our study, treatment-related sentiments were polarized toward either negative or positive—or sometimes both—reflecting trade-offs between efficacy and side effects resulting from tapering-related flares, which were commonly discussed in relation to GCs. Negative sentiments were mostly related to side effects/intolerability, inefficacy/waning efficacy and flare-ups during GC tapering.

A previous survey showed that patients on long-term GCs for rheumatic conditions tended to perceive GCs as effective in managing their condition, with therapeutic benefits outweighing adverse effects [37]. However, our social media data present a more heterogeneous picture, with mixed sentiments about GCs. Flares during tapering were perceived negatively, reflecting the prominent impact of symptoms of active PMR/GCA on patients’ QOL and the ability to take part in everyday activities. Patients expressed concerns about long-term treatment of PMR and GCA, mainly due to potential adverse effects associated with prolonged GC use. However, GCs currently remain the cornerstone of therapy for GCA and PMR, and GC-sparing options remain limited [38].

In our study, the patients’ conversations indicated that emergency room (ER) doctors and general practitioners (GP) were usually the first points of contact for those with PMR and GCA after initial symptoms. Following these initial consultations, patients were often referred to rheumatologists or other specialists such as neurologists or ophthalmologists for confirmation of the diagnosis. The most common unmet need related to HCPs was ‘lack of disease awareness’. In a study that implemented a GP education program on PMR, the time from onset of symptoms to clinic referral was reduced from 42.9 to 24.3 days [39]. However, providing GP education alone might not be sufficient; in this study, a wide range of specialists, including ER doctors, neurologists, ENT doctors, immunologists, orthopaedists and pain management specialists, were involved in the diagnostic journey. It may also be necessary to implement targeted educational outreach to these specialties.

At the time of this study, large language models (LLMs) like ChatGPT were rarely used by patients and physicians. However, they are now being informally adopted by patients and physicians [40]. Optimists suggest that these digital tools might act as ‘copilot’ to suggest differential diagnoses. Nonetheless, the plausibility and fluency of the output of generative artificial intelligence (AI) tools also bring risks of ‘automation bias’ (overreliance on the output), leading to premature diagnostic closure and potentially exacerbating misdiagnosis. There is an urgent need for more research into how LLMs may change the diagnostic process. The use of LLMs may also selectively amplify popular narratives at the expense of minority views; however, the period covered by this study predates the widespread use of AI.

Patients reported both positive and negative experiences with HCPs. Negative experiences included lack of empathy, long waiting periods, delayed diagnosis and misdiagnosis, while positive experiences involved proper attention, care and cooperation.

Patients frequently use social media platforms to share disease experiences. Previous studies based on online communities indicate that patients commonly describe the physical, functional, emotional, cognitive and social implications of their condition [30]. Similarly, in our study, GCA and PMR patients reported that their disease significantly impacted the physical, functional, emotional, financial and social aspects of QOL, resulting in a substantial negative effect on their overall well-being. Patients with PMR complained of pain, fatigue and reduced mobility, and patients with GCA reported fatigue or ‘lack of stamina’ and visual impairment as the main cause of concern. The fear of losing vision is stressful for patients with GCA [41]. In a previous survey, patients with GCA rated ‘losing sight in both eyes permanently’ as the most important QOL domain [42]. Chronic pain limiting patients’ physical and functional life in turn caused depression and impacted the emotional well-being of patients. The unpredictable nature of the disease and difficult steroid-tapering journey caused anxiety among patients, which further impaired their emotional well-being, with patients commonly reporting feeling depressed. Patients were also concerned about the effectiveness of their prescribed treatment and the associated side effects. Furthermore, symptoms such as pain and fatigue impacted daily activities and work productivity.

The current literature on GCA and PMR focuses on balancing control of the disease against treatment-related adverse effects [6]. This social media listening study confirmed that QOL was a central consideration for patients, including living with lasting symptoms such as fatigue, chronic pain and visual impairment and the ability to carry out daily activities or work. These findings suggest that patients with GCA and PMR experience a substantial burden of disease and that there is a need to address these concerns, which could potentially translate into enhancing the QOL of patients and treatment outcomes. Some of the approaches to address these gaps may include personalized care, patient stratification, access to varied diagnostic approaches, development of disease-specific patient-reported outcomes, policy measures and research to characterize the differential burden between the patient subgroups [43].

Currently only limited treatment options are available for PMR and GCA. The insights obtained through our study highlight the unmet needs of patients, particularly concerning the lack of effective and well-tolerated treatment options including GC-sparing agents. Patients with PMR and GCA and their caregivers also highlight the need for greater disease awareness among HCPs. This could aid timely diagnosis and referral, thereby enhancing patient care and outcomes.

Limitations of the study include the impossibility of independently verifying diagnosis in anonymous social media posts. Some of the patients mentioned normal blood test results, negative biopsies or young age. A diagnosis of GCA is difficult to verify without confirmatory tests such as TAB or vascular imaging [44]. Our study is also limited by selection bias. Social media listening studies are likely biased towards the views of more active or confident users of online communities and less able to represent the views of the digitally excluded or the visually impaired. Given that the data analysed in our study largely originated from social media posts based in the USA, readers should be cautious when extrapolating these findings to populations in other geographical regions.

It is essential that HCPs are aware of the commonly discussed themes in online spaces, as the narratives shared, irrespective of medical accuracy, could strongly influence patients’ attitudes towards treatment, as evidenced by the finding that some patients looked online for experiences before initiating treatment. Some social media platforms employ algorithms or other mechanisms to increase the visibility of (‘amplify’) posts that generate more engagement. Users are drawn to stories that resonate with them, creating ‘social media bubbles’. Therefore, the predominance of negative sentiments observed in this study should not necessarily be interpreted as representative of the lived experiences of patients with PMR/GCA in the general population.

In conclusion, the insights obtained through social media listening of patient’s conversations suggest that both PMR and GCA have a major negative impact on numerous aspects of QOL and reveal that patients express a significant unmet need concerning HCPs’ perceived understanding of these diseases, delayed diagnosis and the limited treatment options available. Patient conversations also revealed a need for GC-sparing therapies that are effective in preventing relapses, improve patients’ QOL and are better tolerated.

## Supplementary Material

rkaf140_Supplementary_Data

## Data Availability

The datasets generated or analysed during this study are available from the corresponding author upon reasonable request.
