# Supplementary material for: Patient perspectives on life impact and unmet needs in giant cell arteritis and polymyalgia rheumatica: insights from social media
Source: Rheumatol Adv Pract. 2025 Dec 2;10(1):rkaf140. doi: 10.1093/rap/rkaf140 (PMC12758118; doi:10.1093/rap/rkaf140)
Supplement: rkaf140_Supplementary_Data [file rkaf140_supplementary_data.zip › Supplementary_material (2).docx]

**Supplementary Data S1.**

**Keyword query used for data extraction from the Sprinklr tool for GCA**

("Giant cell arteritis" OR "Temporal arteritis" OR "Arteritis cranialis" OR "cranial arteritis" OR "Arteritis temporalis" OR "GCA")

AND ("Temple pain" OR Tender OR "Inflammation of arteries" OR "Scalp tenderness" OR "Head pain" OR "Jaw pain" OR "Headache" OR "Vision problem" OR "Vision loss" OR "Blurred vision" OR Blind OR Blurry OR "Jaw claudication" OR Fatigue OR Discomfort OR Pain OR "Difficulty chewing" OR "prolonged speaking" OR "Tongue necrosis" OR "Tiredness with chewing" OR Fever OR "Weight loss" OR "Joint pain" OR "muscle pain" OR "Tender scalp" OR "Double vision" OR Myalgia OR Arthralgia OR "scalp necrosis" OR "hearing loss" OR "limb claudication" OR "temporal tenderness" OR Inflammation OR "neck pain" OR Claudication OR "Night sweat" OR "chronic dry cough" OR "Inflammation in arteries" OR stiffness OR "Muscle ache" OR "Mobility issues" OR "Limited motion" OR "Pain in morning" OR "Brain fog" OR "Ocular impairment" OR Flares OR "Disturbance in sleep" OR "difficult sleeping" OR "Flare-ups" OR "Vision changes" OR vasculitis OR "Visual complication" OR Cranial OR bitemporal OR Malaise OR Anaemia OR Chew OR jaw OR hurt OR "Morning stiffness" OR "Shoulder pain" OR "Pelvic girdle pain" OR Arm OR Hip OR "Lower back" OR Buttock OR Thigh OR Wrist OR Elbow OR Knee OR Depression OR "Loss of appetite"

OR "Erythrocyte sedimentation rate" OR "ESR" OR "C-reactive protein" OR "CRP" OR "Colour Doppler ultrasound" OR "CDUS" OR "Magnetic resonance angiography" OR "MRA" OR "Positron emission tomography" OR "PET" OR Biopsy OR "Temporal artery biopsy" OR "TAB" OR "Blood test" OR "Physical exam" OR "Arm strength" OR Ultrasound OR "Magnetic resonance imaging" OR "MRI" OR "CT scan" OR "CT with angiography" OR "CTA"

OR Corticosteroid OR Prednisone OR Methotrexate OR Trexall OR Tocilizumab OR Actemra OR Prednisolone OR Rinvoq OR Glucocorticoids OR Biologic OR Immunosuppressive OR Abatacept OR Steroid OR "Physical therapy" OR Surgery OR "Cold compress" OR NSAIDs OR Kevzara OR Sarilumab OR DMARD OR Cosentyx OR "Novel oral" OR Rituximab OR Adalimumab OR Ustekinumab OR Secukinumab OR Belimumab OR Guselkumab OR Anakinra OR Tremfya OR Kineret OR Mavrilimumab OR Kiniksa OR Stelara OR Orencia OR Olumiant OR Baricitinib OR Upadacitinib OR "Methyl-prednisolone" OR "Methyl prednisolone" OR "Meth prednisolone" OR "Triamcinolone Acetonide" OR Dexamethasone OR Fluticasone OR Propionate OR Hydrocortisone OR "Prednisolone Acetate" OR "Clobetasol Propionate" OR Immunosuppressant OR "Methotrexate Sodium" OR Cyclosporine OR Leflunomide OR Azathioprine OR "Mycophenolate Mofetil" OR Benzonatate

OR "Heart attack" OR Stroke OR "myocardial infarction" OR Aortitis OR "Pulmonary fibrosis" OR Aneurysm OR Diabetes OR Neuropathy OR "Blood pressure" OR "Polymyalgia rheumatica" OR Gastrointestinal OR "GI disease" OR "Ulcerative colitis" OR "UC" OR "Crohn's Disease" OR "Inflammatory bowel disease" OR "CD" OR "IBD" OR "disease of the Oesophagus" OR Oesophagus OR stomach OR duodenum OR "intestine disease" OR "GI perforation" OR Lupus OR Gout OR Sjogren OR "Rheumatoid arthritis" OR "RA" OR "celiac disease" OR " irritable bowel syndrome" OR "IBS" OR Gastroparesis

OR Rheumatologist OR "Primary care physician" OR "General physician" OR "PCP" OR "GP" OR Specialist OR Doctor OR Physiotherapist OR Doc OR Ophthalmologist OR Neurologist OR Internist OR Geriatrics)

**Keyword query used for data extraction from the Sprinklr tool for GCA**

("Polymyalgia rheumatica" OR "Polymyalgiarheumatica" OR "Polymy rheumatica" OR "Polymyalg rheumatica" OR "PMR")

AND ("Temple pain" OR Tender OR "Inflammation of arteries" OR "Scalp tenderness" OR "Head pain" OR "Jaw pain" OR "Headache" OR "Vision problem" OR "Vision loss" OR "Blurred vision" OR Blind OR Blurry OR "Jaw claudication" OR Fatigue OR Discomfort OR Pain OR "Difficulty chewing" OR "prolonged speaking" OR "Tongue necrosis" OR "Tiredness with chewing" OR Fever OR "Weight loss" OR "Joint pain" OR "muscle pain" OR "Tender scalp" OR "Double vision" OR Myalgia OR Arthralgia OR "scalp necrosis" OR "hearing loss" OR "limb claudication" OR "temporal tenderness" OR Inflammation OR "neck pain" OR Claudication OR "Night sweat" OR "chronic dry cough" OR "Inflammation in arteries" OR stiffness OR "Muscle ache" OR "Mobility issues" OR "Limited motion" OR "Pain in morning" OR "Brain fog" OR "Ocular impairment" OR Flares OR "Disturbance in sleep" OR "difficult sleeping" OR "Flare-ups" OR "Vision changes" OR vasculitis OR "Visual complication" OR Cranial OR bitemporal OR Malaise OR Anaemia OR Chew OR jaw OR hurt OR "Morning stiffness" OR "Shoulder pain" OR "Pelvic girdle pain" OR Arm OR Hip OR "Lower back" OR Buttock OR Thigh OR Wrist OR Elbow OR Knee OR Depression OR "Loss of appetite"

OR "Erythrocyte sedimentation rate" OR "ESR" OR "C-reactive protein" OR "CRP" OR "Colour Doppler ultrasound" OR "CDUS" OR "Magnetic resonance angiography" OR "MRA" OR "Positron emission tomography" OR "PET" OR Biopsy OR "Temporal artery biopsy" OR "TAB" OR "Blood test" OR "Physical exam" OR "Arm strength" OR Ultrasound OR "Magnetic resonance imaging" OR "MRI" OR "CT scan" OR "CT with angiography" OR "CTA" OR "WORC index" OR "Western Ontario Rotator Cuff (WORC) Index" OR "WORC-Index"

OR Corticosteroid OR Prednisone OR Methotrexate OR Trexall OR Tocilizumab OR Actemra OR Prednisolone OR Rinvoq OR Glucocorticoids OR Biologic OR Immunosuppressive OR Abatacept OR Steroid OR "Physical therapy" OR Surgery OR "Cold compress" OR NSAIDs OR Kevzara OR Sarilumab OR DMARD OR Cosentyx OR "Novel oral" OR Rituximab OR Adalimumab OR Ustekinumab OR Secukinumab OR Belimumab OR Guselkumab OR Anakinra OR Tremfya OR Kineret OR Mavrilimumab OR Kiniksa OR Stelara OR Orencia OR Olumiant OR Baricitinib OR Upadacitinib OR "Methyl-prednisolone" OR "Methyl prednisolone" OR "Meth prednisolone" OR "Triamcinolone Acetonide" OR Dexamethasone OR Fluticasone OR Propionate OR Hydrocortisone OR "Prednisolone Acetate" OR "Clobetasol Propionate" OR Immunosuppressant OR "Methotrexate Sodium" OR Cyclosporine OR Leflunomide OR Azathioprine OR "Mycophenolate Mofetil" OR Benzonatate

OR "Heart attack" OR Stroke OR "myocardial infarction" OR Aortitis OR "Pulmonary fibrosis" OR Aneurysm OR Diabetes OR Neuropathy OR "Blood pressure" OR "Giant cell arteritis" OR "Temporal arteritis" OR "GCA" OR "Arteriitis cranialis" OR "cranial arteritis" OR "Arteriitis temporalis" OR Gastrointestinal OR "GI disease" OR "Ulcerative colitis" OR "UC" OR "Crohn's Disease" OR "Inflammatory bowel disease" OR "CD" OR "IBD" OR "disease of the Oesophagus" OR Oesophagus OR stomach OR duodenum OR "intestine disease" OR "GI perforation" OR Lupus OR Gout OR Sjogren OR "Rheumatoid arthritis" OR "RA" OR "celiac disease" OR "irritable bowel syndrome" OR "IBS" OR Gastroparesis

OR Rheumatologist OR "Primary care physician" OR "General physician" OR "PCP" OR "GP" OR Specialist OR Doctor OR Physiotherapist OR Doc OR Ophthalmologist OR Orthopaedics OR "ER" OR Geriatrics)
